# Factors Associated with COVID-19 Vaccine Intentions among South Carolina Residents

**DOI:** 10.3390/vaccines10060942

**Published:** 2022-06-14

**Authors:** Mufaro Kanyangarara, Lauren McAbee, Virginie G. Daguise, Melissa S. Nolan

**Affiliations:** 1 Department of Epidemiology and Biostatistics, University of South Carolina, Columbia, SC 29208, USA; lgmcabee@email.sc.edu (L.M.); msnolan@mailbox.sc.edu (M.S.N.); 2 South Carolina Department of Health and Environmental Control, Columbia, SC 29201, USA; daguisvg@dhec.sc.gov

**Keywords:** COVID-19, vaccination, vaccine intentions, South Carolina, United States

## Abstract

Despite evidence of vaccine safety and efficacy, vaccine hesitancy remains a major global health threat. The COVID-19 vaccine has presented unique vaccine hesitancy concerns compared to parental vaccine hesitancy towards childhood vaccines. South Carolina (SC) is home to a largely conservative population and historically has some of the lowest vaccination coverage rates in the United States of America. The goal of the current study was to identify factors associated with COVID-19 vaccine intentions among SC residents. From November 2020 to September 2021, 300,000 invitations to participate in community testing and complete an online survey were mailed to randomly selected SC residents. The survey collected data about behaviors and attitudes towards COVID-19 vaccines, as well as demographic and health characteristics. Of the 10,626 survey participants, 69.9% reported being vaccinated against COVID-19. Among those not vaccinated, 65.5% reported vaccine intentions. Logistic regression analyses were performed to examine factors associated with COVID-19 vaccine intentions. Multivariate logistic regression results indicated that confidence in the safety of the COVID-19 vaccines increased the likelihood of vaccine intentions, while younger age (<60 years) decreased the likelihood of vaccine intentions. To increase vaccine intentions and uptake, public health and government officials in South Carolina and other conservative states should target younger populations and address concerns about COVID-19 vaccine safety.

## 1. Introduction

To date, the COVID-19 pandemic has resulted in over 533 million cases and caused over 6.3 million deaths globally. In the United States (US), there have been over 85 million cases and 1 million deaths [1]. Vaccines are among the best tools to prevent the spread of COVID-19 and address the pandemic. Currently, the Center for Disease Control and Prevention (CDC) recommends individuals 5 years of age and older receive their primary series of a COVID-19 vaccine [2]. As of May 2022, over 255 million people (77.8%) in the US have received at least one dose of a COVID-19 vaccine, and over 220 million people (66.4%) are fully vaccinated against COVID-19 [1]. To reach herd immunity, it is estimated that at least 70% of the population needs to be vaccinated [3]. Addressing vaccine hesitancy is crucial in ending the pandemic and ensuring that herd immunity is reached.

Vaccine hesitancy is defined by the World Health Organization as “a reluctance or refusal to vaccinate despite the availability of vaccines” and is considered one of the top 10 global health threats [4]. Drivers and barriers of vaccination consist of a myriad of factors including age, gender, socioeconomic status, education level, attitudes regarding the vaccines, access to vaccines, lack of trust in the vaccines, misinformation, and misconceptions about the vaccines [5,6,7,8,9]. Prior to the COVID-19 pandemic, vaccine hesitancy research was principally focused on routine childhood, influenza, and human papillomavirus (HPV) vaccines [9]. Across the US, non-medical vaccination exemption rates have steadily increased in recent years, with 18 states permitting non-medical exemptions for school enrollment and up to 27% of some pediatric populations are still unvaccinated by the age of 5 years [10]. This insidious anti-vaccine movement has resulted in multiple epidemics including a measles epidemic that hit an all-time high of more than 1200 cases just as the COVID-19 pandemic began [11,12]. Emerging evidence assessing the impact of the COVID-19 pandemic on routine childhood vaccines suggests that, while vaccine hesitancy has been on the rise, vaccine acceptance remains unchanged [13].

The COVID-19 mRNA vaccine presents novel public health challenges, as this is the first time that large segments of the adult population have refused vaccination. The pandemic continues to underscore the significant role of medical mistrust and government misinformation in driving vaccine hesitancy and the need to understand factors driving vaccine intentions and uptake. The goal of this paper was to assess factors associated with COVID-19 vaccine intentions among South Carolina (SC) residents. This was a secondary data analysis of data collected as a part of the SC Sampling and Testing Representative Outreach for Novel coronavirus Guidance (SC STRONG) project. The project was led by the South Carolina Department of Health and Environmental Control (SCDHEC) and the University of South Carolina (UofSC). SC STRONG was established in October 2020, with the goal of assessing population-level prevalence of COVID-19 across SC and examining knowledge, attitudes, and behaviors surrounding COVID-19 transmission. This study uses information gathered from the SC STRONG project to identify factors associated with COVID-19 vaccine intentions among SC residents. In the US, the south has the highest rates of vaccine hesitancy and the lowest vaccination rates [14]. A systematic review found that COVID-119 vaccine hesitancy was more prevalent among women and African Americans, as well as unemployed, lower income, lower education, and younger age individuals [15]. While several studies have evaluated vaccine knowledge, intentions, and uptake, few have considered SC, where vaccine coverage is low and transmission of COVID-19 high [15,16,17]. Given the heterogeneity of vaccine acceptance across geographic regions, this study fulfills a gap in research for factors influencing COVID-19 vaccine intentions in SC. While vaccine intentions may not always be reflective of vaccine uptake, understanding the factors associated with vaccine intentions will help officials to target interventions toward vaccine-hesitant populations and increase vaccine uptake.

## 2. Materials and Methods

### 2.1. Study Setting

SC has a population of about 5.1 million people, about 18% of the population is 65 years or older, and about 52% of the population is female [18]. The majority of the population is either white (69%) or black (27%) [18]. As of May 2022, there have been over 1.48 million confirmed cases of COVID-19 and over 17,800 confirmed deaths due to COVID-19 in SC [19]. About 63% of eligible SC residents have had at least one dose of a COVID-19 vaccine, and 54% of eligible SC residents are fully vaccinated against COVID-19 [19]. SC also remains among the lowest ranked states when comparing the share of the population that is fully vaccinated [1].

### 2.2. Study Procedure

All SC residents over the age of 5 years were eligible to participate in the SC STRONG project. According to the U.S. Census Bureau, only about 5.7% of SC’s population is children under 5 years old [18], so most SC residents were eligible. The SC STRONG project consisted of four phases: 4 November 2020 to 31 December 2020, 1 February 2021 to 4 March 2021, 1 May 2021 to 28 June 2021, and 1 August 2021 to 30 September 2021. During each phase, SC residents were randomly selected, using the World Health Organization (WHO) modified cluster sampling. The final target sample size was 6500 participants. Randomly selected residents were mailed a paper-based invitation to participate in an online survey and test for COVID-19 infection and antibodies as part of the SC STRONG project. Of the 750,063 residents randomly selected and invited to participate in the SC STRONG project, the overall survey response rate was 2.0%. Further details discussing the objectives and methods of the SC STRONG project can be found elsewhere [20].

Surveys were sent to participants to assess factors that may influence vaccine intentions. Some questions in the surveys included attitudes and behaviors regarding COVID-19 and the vaccines, motivators, and barriers to getting vaccinated, and demographic characteristics. The questionnaire was completely online and was administered through REDCap. The questionnaire consisted of questions regarding occupation, exposures to SARS-CoV-2, perceptions of risk regarding COVID-19 infection, and opinions on COVID-19 vaccines (Supplementary File Table S1). Questions regarding the COVID-19 vaccines were not added to the survey until January 2021 after the first COVID-19 vaccines became available in SC. Of the 14,804 survey responses, 10,626 with data on COVID-19 vaccine intentions and uptake were included.

### 2.3. Statistical Analysis

Sociodemographic characteristics including age, gender, and race/ethnicity were described using descriptive statistics. The outcome of interest was intention to get vaccinated against COVID-19. Factors of interest for the analysis included age, race, yearly household income level, underlying health conditions, concerns about COVID-19, and opinions on the COVID-19 vaccines. Univariate and multivariate logistic regression analyses were conducted to identify factors associated with COVID-19 vaccine intentions. Backwards elimination was used to retain statistically significant variable (*p* < 0.05) in the multivariate model. Unadjusted and adjusted odds ratios and 95% confidence intervals were presented as measures of association. STATA BE/17.0 software was used for all analyses. A *p*-value of 0.05 was considered significant.

### 2.4. Ethical Considerations

Participation in the SC STRONG project, both testing and survey completion, was completely voluntary. Ethical approval for the SC STRONG project was sought and obtained from the Institutional Review Boards (IRBs) at the University of South Carolina (UofSC) and SCDHEC.

## 3. Results

Most participants were 60 years of age or older (60.1%), female (55.8%), white (83.3%), and reported an income below USD 100,000 (45.3%) (Table 1). The most common underlying health condition was hypertension (35.0%), followed by obesity (13.4%). Most participants had been tested for COVID-19 before (64.1%), and 22.5% of those with a history of COVID-19 had received a positive COVID-19 test result. Most participants agreed that the COVID-19 vaccines were safe (75.4%) and effective (76.0%). Among the 69.9% of participants who reported being vaccinated against COVID-19, the most common motivators for vaccination were protecting themselves (78.0%), doing their part to help control the pandemic (71.2%), and protecting high-risk friends and family (59.5%). Most vaccinated individuals were 60 years or older (68.1%), 24.6% were 40–59 years, and 7.3% were younger than 40 years. One notable difference between vaccinated and unvaccinated individuals was regarding the safety and efficacy of the vaccines. Most vaccinated individuals felt that the vaccines were safe (88.3%) and effective (88.0%), but less than half of the unvaccinated participants had confidence in the safety (45.4%) and effectiveness (48.1%) (Table 1).

Of the unvaccinated, 65.5% reported intentions to vaccinate. Most of those who had intentions to get vaccinated against COVID-19 were 60 years or older (45.1%), female (53.2%), thought the vaccines were safe (68.4%) or effective (68.2%), and knew a close friend or family that had tested positive for COVID-19 (71.3%) (Table 2). Those without vaccine intentions tended to be 40–59 years (45.6%), female (56.5%), and residing in a household with an income between USD 50,000 and 99,999 (26.5%). Vaccine intentions decreased with time, with the highest vaccine intentions among respondents surveyed in February and March 2021 (85.4%) and the lowest among respondents surveyed in August and September 2021 (32.7%).

**Figure 1:**
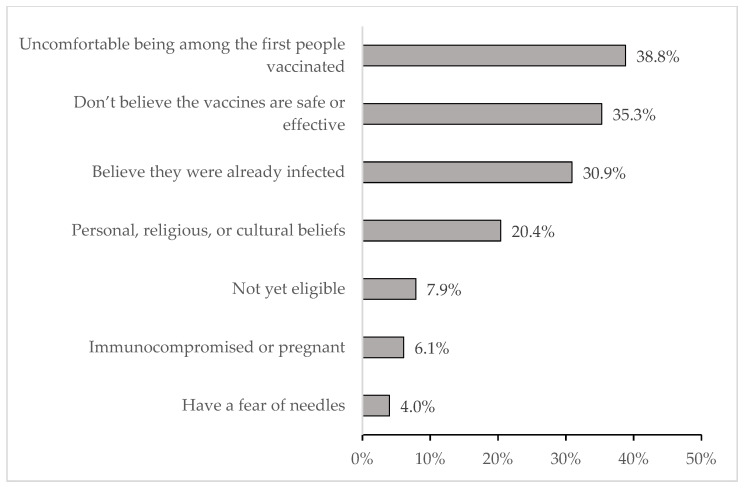
Barriers to getting vaccinated among those who do not have vaccine intentions (*n* = 1054) *. * Multiple responses were permitted.

Among those with no vaccine intentions, the most common barriers to getting vaccinated were not believing that the vaccines were safe and effective (35.5%), not believing that they needed the vaccine because they had a previous COVID-19 infection (30.9%), and not being comfortable being among the first people vaccinated (38.8% (Figure 1)). Having a fear of needles was the least common barrier, with only 4.0% of participants indicating that choice.

From the univariate analysis, those who felt the vaccines were safe were about 35 times more likely to have intentions to get vaccinated (OR 34.75, 95% CI 26.26–45.99), and those who thought the vaccines were effective 13 times more likely (OR 13.41, 95% CI 10.95–16.43) (Table 3). Those who were concerned or very concerned about themselves or their household getting infected with COVID-19 were about 9 times more likely to have vaccine intentions (OR 9.73, 95% CI 7.75–12.22), and those who were concerned or very concerned about the spread of COVID-19 in the community were more likely to have vaccine intentions (OR 14.93, 95% CI 11.49–19.41). Those who were surveyed after phase 2 were less likely to have vaccine intentions (phase 3: OR 0.11, 95% CI 0.09–0.15; phase 4: OR 0.08, 95% CI 0.07–0.10).

The results of the multivariate analysis indicated that those who believed the COVID-19 vaccines were safe were significantly more likely to have vaccine intentions than those who did not believe that COVID-19 vaccines were safe (Table 3). Participants who felt that the vaccines were safe were 13 times more likely to have intentions to get vaccinated (aOR 13.0, 95% CI 9.25–18.38) compared to participants who did not. Those who felt vaccines were effective were about 3 times more likely to have intentions compared to those who did not agree (aOR 2.83, 95% CI 2.12–3.77). Those who were a little concerned (aOR 1.88, 95% CI 1.35–2.61) or concerned/very concerned (aOR 2.63 95% CI 1.80–3.85) about themselves or their household getting infected with COVID-19 were also more likely to have vaccine intentions, as were those who were a little concerned (aOR 2.78, 95% CI 1.91–4.05) or concerned/very concerned (aOR 4.83, 95% CI 3.19–7.31) about the spread of COVID-19 in their communities. The multivariate analysis also showed that those who were younger than 60 years had a significantly lower likelihood of having vaccine intentions. Those who were obese (aOR 0.53, 95% CI 0.34–0.81) or had previously tested positive for COVID-19 (aOR 0.71, 95% CI 0.55–0.90) were also less likely to have vaccine intentions.

## 4. Discussion

This study examined factors associated with COVID-19 vaccine intentions among SC residents. About two-thirds (69.9%) of participants were already vaccinated, and most (65.5%) of the unvaccinated had vaccine intentions. The most notable finding of this study was that those who felt the vaccines were safe were 13 times more likely to have vaccine intentions, compared to their counterparts. This finding is consistent with other studies, where a lack of confidence in vaccine safety was found to be associated with lower odds of getting vaccinated [21,22,23]. Concerns about the safety of the vaccines are very impactful on vaccine intentions and may be due to a lack of trust with the medical community [24]. The role of health care professionals is key in increasing vaccine uptake, but lack of trust in health care providers may hinder vaccine acceptance. Concerns about vaccine safety also may come from misinformation regarding the vaccines. Individuals who believe that vaccines are unsafe are more likely to believe vaccine myths and less likely to get vaccinated [23]. Vaccine misinformation can quickly spread across social media, and it threatens vaccine acceptance [25,26]. Thus, it is important to emphasize accurate information about the safety and efficacy of the vaccines to support vaccine uptake and herd immunity.

The present study also found that those who were concerned about COVID-19 infection were more likely to have vaccine intentions. Perceived susceptibility and perceived risk are important factors to decision making in regard to vaccination, and perceived susceptibility is affected by income level, certain health conditions, and types of transportation [27]. Attitudes surrounding vaccines are very influential in vaccine hesitancy or acceptance [28], so it is important to educate on susceptibility to COVID-19 and the importance of vaccination.

In addition to vaccine safety and concerns about getting infected, age was also a significant predictor of COVID-19 vaccine intentions in the present study. Several studies have shown that age strongly influences vaccine intentions [3,5]. Younger people may be less likely to have intentions of getting vaccinated because they may not believe that COVID-19 could affect their health, and low perceived susceptibility may affect vaccine intentions [27]. Low perceived susceptibility among younger people could be due to plethora of information centered around COVID-19 and the risk in older people. Thus, younger people may not see the importance in getting vaccinated. Older people are much more likely to suffer serious health consequences due to COVID-19, and at the beginning of the pandemic, they were much more likely to practice COVID-19 prevention measures [29]. The resurgent COVID-19 epidemics across the US have been attributed younger populations, yet uptake in the population remains low [7]. Informing younger people about the truths of COVID-19 and how it can negatively affect everyone is important in ensuring vaccine uptake.

There are a several limitations worth noting. First, the cross-sectional nature of survey precludes any causal inferences. Although the sampling method employed attempted to ensure sample representativeness, the findings presented may not be representative of SC. As an online survey, internet access was needed to complete the survey. The response rate was only 2%; therefore, respondents may not be representative of the entire SC population. The sociodemographic characteristics of survey respondents were similar to those of the general SC population on some characteristics; however, there were differences by race and age. For instance, Hispanics/Latin Americans and African Americans were underrepresented, while older adults (>60 years) were overrepresented. Due to social desirability bias, survey responses may not be indicative of true intentions regarding COVID-19 vaccines. Additionally, reported vaccine intentions may not represent future vaccine acceptance and uptake. Intentions have often been used as a proxy for behavior; however, it is possible vaccine intentions may not be reflective of uptake, especially as the pandemic progresses. The changing burden of COVID-19, availability of COVID-19 vaccines, rise of new information about the disease and vaccines, and the emergence of COVID-19 variants may influence vaccine intentions and uptake. Indeed, findings from this study suggest the likelihood of vaccine intentions decreased with time. Lastly, data on several factors that influence vaccine intentions were not collected. Especially in the south of the US, political opinions and religious beliefs continue to influence health behaviors and motivations [30]. Given the politicization of public health measures to reduce the transmission of COVID-19, inclusion of such information would have shed more light on vaccine intentions.

## 5. Conclusions

Despite the limitations, this study leveraged a large sample of SC residents to examine vaccine intentions across the entire state. The results of this study show a strong need to target specific populations in SC to potentially increase COVID-19 vaccine uptake. In recent months, vaccination coverage rates have stagnated. The findings from this study can inform the development of strategies to reach the unvaccinated population in SC and increase vaccination coverage.

## Figures and Tables

**Table 1:** Characteristics of participants in South Carolina, categorized by vaccination status.

Characteristic	Total Population (*n* = 10,626) *n* (%)	Vaccinated (*n* = 7432) *n* (%)	Unvaccinated (*n* = 3194) *n* (%)	*p*-Value
Age Range				<0.001
<40 years	1090 (10.5)	529 (7.3)	561 (18.3)	
40–59 years	3043 (29.4)	1795 (24.6)	1248 (40.6)	
60+ years	6233 (60.1)	4970 (68.1)	1263 (41.1)	
Gender				<0.001
Female	5927 (55.8)	4182 (56.7)	1745 (54.6)	
Male	4383 (41.3)	3081 (41.5)	1302 (40.8)	
Other or prefer not to say	316 (3.0)	169 (2.3)	147 (4.6)	
Race/Ethnicity *				
Hispanic/Latinx	154 (1.5)	95 (1.3)	59 (1.8)	0.5
White/Caucasian	8856 (83.3)	6345 (85.4)	2511 (78.6)	0.002
Black/African American	718 (6.8)	449 (6.0)	269 (8.4)	0.2
Behaviors and Attitudes				
Think COVID-19 vaccines are safe	7988 (75.4)	6545 (88.3)	1443 (45.4)	<0.001
Think COVID-19 vaccines are effective	8040 (76.0)	6512 (88.0)	1528 (48.1)	<0.001
Know friends or family who have had COVID-19	7522 (70.8)	5161 (69.4)	2.361 (73.9)	<0.001
Been tested for COVID-19	6807 (64.1)	4729 (63.7)	2078 (65.1)	0.2
Tested positive for COVID-19	1527 (22.5)	858 (18.2)	669 (32.3)	<0.001
Been tested for COVID-19 antibodies	1537 (14.5)	1137 (15.3)	400 (12.6)	<0.001
Tested positive for COVID-19 antibodies	423 (27.9)	299 (26.7)	124 (31.3)	0.08
Levels of concern about household or self getting infected with COVID-19				<0.001
Concerned/very concerned	5026 (47.9)	3548 (48.2)	1478 (47.2)	
A little concerned	3866 (36.9)	2787 (37.9)	1079 (34.4)	
Not concerned	1598 (15.2)	1020 (13.9)	578 (18.4)	
Levels of concern about spread of COVID-19 in community				<0.001
Concerned/very concerned	6901 (65.8)	5104 (69.4)	1797 (57.3)	
A little concerned	2725 (26.0)	1817 (24.7)	908 (29.0)	
Not concerned	865 (8.3)	435 (5.9)	430 (13.7)	
Health Problems				
Hypertension	3721 (35.0)	2823 (38.0)	898 (28.1)	<0.001
Obesity	1427 (13.4)	1264 (17.0)	163 (5.1)	<0.001
Diabetes	1210 (11.4)	935 (12.6)	275 (8.6)	<0.001
Asthma	893 (8.4)	642 (8.6)	251 (7.9)	0.2
Heart disease	829 (7.8)	673 (9.1)	156 (4.9)	<0.001
Immunocompromising condition	559 (5.3)	428 (5.8)	131 (4.1)	<0.001
Lung disease	393 (3.7)	291 (3.9)	102 (3.2)	0.07
Blood clotting disorder	177 (1.7)	114 (1.5)	63 (2.0)	0.1
Work				
Essential worker	1696 (16.0)	1054 (14.2)	642 (20.1)	<0.001
Front-line medical care worker	660 (6.2)	548 (7.4)	112 (3.5)	<0.001
Works at nursing home/rehab center/long-term care facility	91 (0.9)	70 (0.9)	21 (0.7)	0.1
Household Income				<0.001
Less than USD 50,000	1852 (17.4)	1219 (16.4)	633 (19.8)	
USD 50,000–99,999	2960 (27.9)	2066 (27.8)	894 (28.0)	
USD 100,000 or more	3023 (28.5)	2194 (29.5)	829 (26.0)	
Motivators to getting vaccinated				
Protecting themselves	8287 (78.0)	6664 (89.7)	1623 (50.8)	<0.001
Doing their part to control pandemic	7569 (71.2)	6042 (81.3)	1527 (47.8)	<0.001
Protecting high-risk friends/family	6317 (59.5)	4947 (66.6)	1370 (42.9)	<0.001
Concerned about exposure in community	6077 (57.2)	4930 (66.3)	1147 (35.9)	<0.001
Serve as example for others to get vaccinated	5080 (47.8)	4159 (56.0)	921 (28.8)	<0.001
Concerned about exposure at work/school	3273 (30.8)	2513 (33.8)	760 (23.8)	<0.001

* Those who selected Asian, Native American, other, or selected more than one race/ethnicity were not included in this table.

**Table 2:** Characteristics of unvaccinated participants, categorized by intent to vaccinate. (*n* = 3056) ^†^.

Characteristic	Intend to Vaccinate (*n* = 2002) *n*(%)	Do not intend to Vaccinate (*n* = 1054) *n*(%)	*p*-Value
Age Range			<0.001
<40 years	329 (17.0)	217 (21.6)	
40–59 years	736 (38.0)	457 (45.6)	
60+ years	874 (45.1)	329 (32.8)	
Gender			0.03
Female	1064 (53.2)	595 (56.5)	
Male	857 (42.8)	403 (38.2)	
Other or prefer not to say	81 (4.1)	56 (5.3)	
Race/Ethnicity *			
Hispanic/Latinx	42 (2.2)	16 (1.7)	0.1
White/Caucasian	1589 (83.8)	825 (86.0)	0.8
Black/African American	186 (9.8)	68 (7.1)	0.002
Behaviors and Attitudes			
Think COVID-19 vaccines are safe	1365 (68.4)	61 (5.8)	<0.001
Think COVID-19 vaccines are effective	1358 (68.2)	141 (13.4)	<0.001
Know friends or family who have had COVID-19	1428 (71.3)	827 (78.5)	<0.001
Been tested for COVID-19 antibodies	253 (12.7)	127 (12.1)	0.7
Been tested for COVID-19	1251 (62.6)	730 (69.3)	<0.001
Tested positive for COVID-19 antibodies	59 (23.6)	59 (46.8)	<0.001
Tested positive for COVID-19	292 (23.4)	335 (46.1)	<0.001
Levels of concern about household or self getting infected with COVID-19			<0.001
Concerned/very concerned	1163 (59.1)	254 (24.6)	
A little concerned	627 (31.8)	400 (38.8)	
Not concerned	179 (9.1)	378 (36.6)	
Levels of concern about spread of COVID-19 in community			<0.001
Concerned or very concerned	1420 (72.1)	309 (29.9)	
A little concerned	451 (22.9)	406 (39.3)	
Not concerned	99 (5.0)	317 (30.7)	
Health Problems			
Asthma	165 (8.2)	75 (7.1)	0.3
Blood clotting disorder	35 (1.8)	23 (2.2)	0.4
Diabetes	200 (10.0)	66 (6.3)	0.001
Heart disease	110 (5.5)	43 (4.1)	0.09
Hypertension	625 (31.2)	234 (22.2)	<0.001
Immunocompromising condition	84 (4.2)	38 (3.6)	0.4
Lung disease	83 (4.2)	18 (1.7)	<0.001
Obesity	61 (3.1)	84 (8.0)	<0.001
Other	247 (12.3)	104 (9.9)	0.04
Work			
Works at nursing home/rehab center/long-term care facility	10 (0.5)	11 (1.0)	0.08
Front-line medical care worker	48 (2.4)	60 (5.7)	<0.001
Essential worker	359 (17.9)	256 (24.3)	<0.001
Household Income			<0.001
Less than USD 50,000	402 (20.1)	203 (19.3)	
USD 50,000–99,999	575 (28.7)	279 (26.5)	
USD 100,000 or more	585 (29.2)	228 (21.6)	
Survey phase			<0.001
1: 4 November–31 December 2020 ^#^	0	0	
2: 1 February–4 March 2021	1522 (85.4)	261 (14.6)	
3: 1 May–28 June 2021	167 (40.1)	250 (59.9)	
4: 1 August–30 September 2021	235 (32.7)	484 (67.3)	

^†^ Due to missing data, those who answered their plan for vaccination does not equal the total unvaccinated in Table 1. * Those who selected Asian, Native American, other, or selected more than one race/ethnicity were not included in this table. ^#^ Questions related to COVID-19 vaccines were added after January 2021, therefore data on vaccine intentions were not available for individuals surveyed in phase 1.

**Table 3:** Factors influencing vaccine intentions for participants in South Carolina. (*n* = 2197).

Factors	Univariate			Multivariate		
	OR	95% CI	*p*-Value	aOR	95% CI	*p*-Value
Age Range						
<40 years	0.57	0.46–0.71	<0.001	0.56	0.42–0.75	<0.001
40–59 years	0.61	0.51–0.72	<0.001	0.68	0.54–0.87	0.002
60+ years	Ref			Ref		
Gender						
Female	Ref			Ref		
Male	1.19	1.02–1.39	0.03	1.13	0.91–1.40	0.3
Other or prefer not to say	1.68	0.66–4.25	0.3	6.62	2.10–20.86	0.001
Race/Ethnicity						
Hispanic/Latinx	1.33	0.74–2.39	0.3			
White/Caucasian	0.99	0.81–1.21	0.9			
Black/African American	1.46	1.10–1.96	0.01	1.38	0.95–1.99	0.09
Behaviors and Attitudes						
Think COVID-19 vaccines are safe	34.75	26.26–45.99	<0.001	13.0	9.25–18.38	<0.001
Think COVID-19 vaccines are effective	13.41	10.95–16.43	<0.001	2.83	2.12–3.77	<0.001
Been tested for COVID-19 antibodies	1.09	0.86–1.37	0.5			
Been tested for COVID-19	0.73	0.62–0.86	<0.001			
Know friends or family who have had COVID-19	0.66	0.55–0.80	<0.001			
Tested positive for COVID-19 antibodies	0.50	0.34–0.73	<0.001			
Tested positive for COVID-19	0.37	0.31–0.45	<0.001	0.71	0.55–0.90	0.006
Levels of concern about household or self getting infected with COVID-19						
Not concerned	Ref			Ref		
A little concerned	3.32	2.67–4.15	<0.001	1.88	1.35–2.61	<0.001
Concerned/very concerned	9.73	7.75–12.22	<0.001	2.63	1.80–3.85	<0.001
Levels of concern about spread of COVID-19 in community						
Not concerned	Ref			Ref		
A little concerned	3.63	2.78–4.74	<0.001	2.78	1.91–4.05	<0.001
Concerned/very concerned	14.93	11.49–19.41	<0.001	4.83	3.19–7.31	<0.001
Health Problems						
Asthma	1.12	0.84–1.49	0.4			
Blood clotting disorder	0.78	0.46–1.33	0.4			
Diabetes	1.61	1.21–2.16	0.001			
Heart disease	1.32	0.92–1.89	0.1			
Hypertension	1.54	1.29–1.84	<0.001			
Immunocompromising condition	1.14	0.77–1.68	0.5			
Lung disease	2.45	1.46–4.10	0.001			
Obesity	0.36	0.25–0.50	<0.001	0.53	0.34–0.81	0.004
Other	1.28	1.00–1.63	0.05			
Work						
Works at nursing home/rehab center/long-term care facility	0.47	0.20–1.10	0.08			
Front-line medical care worker	0.40	0.27–0.59	<0.001			
Essential worker	0.67	0.55–0.80	<0.001			
Household Income						
Less than USD 50,000	Ref					
USD 50,000–99,999	1.06	0.85–1.32	0.6			
USD 100,000 or more	1.30	1.03–1.63	0.03			
Survey Phase						
2: 1 February–4 March 2021	Ref					
3: 1 May–28 June 2021	0.11	0.09–0.15	<0.001			
4: 1 August– 30 September 2021	0.08	0.07–0.10	<0.001			

OR: odds ratio, aOR: adjusted odds ratio, CI: confidence interval, Ref: reference.

## Data Availability

The data presented in this study are available on reasonable request from the corresponding author.

## Supplementary Materials

The following supporting information can be downloaded at: https://www.mdpi.com/article/10.3390/vaccines10060942/s1, Table S1: Sampling and Testing Representative Outreach for Novel coronavirus Guidance.
